# Lectures on Military Surgery

**Published:** 1861-10

**Authors:** E. Andrews

**Affiliations:** Professor of Surgery, Surgeon to the Mercy Hospital; to the Chicago Dispensary, etc.


### CHICAGO MEDICAL EXAMINER

N. S. DAVIS, M. D., Editor.---FRANK W. REILLY, M. D., Ass't Editor.

VOL. II.] OCTOBER, 1861. [NO. 10.

### Original Contributions.

### ARTICLE I.

### LECTURES ON MILITARY SURGERY:

DELIVERED DURING THE SUMMER COURSE OF THE MEDICAL DEPARTMENT OF LIND UNIVERSITY.

By E. ANDREWS, A. M., M. D.,

PROFESSOR OF SURGERY; SURGEON TO THE MERCY HOSPITAL; TO THE CHICAGO DISPENSARY, ETC.

### LECTURE SIXTH.

### HOSPITAL GANGRENE.

When a large number of wounded are placed together in hospital, without sufficient ventilation, or careful dressing, the most disastrous results speedily ensue. Hospital gangrene and traumatic erysipelas break out in an epidemic form, and make fearful havoc among the patients.

The discharges from gun-shot wounds are generally copious, and often offensive, from the presence of gangrenous tissue in the wound. If, therefore, the number of patients is too great for the supply of attendants, the pus will inevitably run down upon the bedding, and by decomposition become horribly foul and offensive. If, in addition to this, the hospital is overcrowded and insufficiently ventilated, the men are constantly bathed in a foul and poisonous atmosphere. Experiment shows that, not only is the air full of ammonia, sulphuretted hydrogen, and other gaseous products of putrefaction, but also is charged with solid particles, such as dried pus-corpuscles, microscopic shreds of dead and dessicated tissues, and excretions. The inhalation from day to day of such an atmosphere, speedily changes the diathesis of a patient from a healthy to a feeble aplastic one, which is ready to take on any kind of disease of the asthenic type. At the same time, either the chemical decomposition of dead animal matter, or else the depraved action of living tissue gives origin to a specific poison capable of being wafted on the air of the hospital, and carried from patient to patient, and spreading the terrible hospital gangrene. This disease generally manifests itself on the edges of a wound, in the form of a small gray or dark gelatinous looking slough of a semi-transparent appearance, as though it were soft, and capable of being wiped away; if however, it is touched with a sponge for the purpose, it is found adherent and possessed of sufficient consistence to withstand the effort. Sometimes the disease begins as a dusky red swelling, like a boil, with a pustule upon it, which breaks and becomes the starting point of the gangrene. The affected part becomes painful. The wound is at first dry and irritated, but afterwards discharges copiously an offensive, unhealthy pus. The edges are apt to be puffed up with a red, erysipelatous looking inflammation, suggesting the idea that the poison present is the same as that of erysipelas.

On the second day the slough will be found enlarged, and it continues to spread in many instances, without any definite limitation, until death. The patient has a low adynamic fever, and dies with symptoms resembling typhus.

When the disease makes its appearance in a wound, it is speedily spread by infection to other patients, and in a short time makes a fearful destruction of life.

The most important part of the treatment in this disease, is prophylactic. It will be almost useless for the surgeon to exhibit particular remedies to the cases as they occur, if he allows the constitution of his men to be undermined by the breathing of poisonous effluviæ. The first thing, therefore, to be seen to, is ventilation and cleanliness.

Ventilation is to be accomplished by proper construction of the hospital. This should be such as to allow, at least, one thousand cubic feet of air to each patient. There should be a very free circulation of air by openings in the sides of the building, near the ground, and others above near the eaves. The foul air will then readily escape at the upper openings, and fresh enter below. If a private house is taken for a temporary hospital, the windows and doors will often suffice for openings; but if there is any doubt about it, large apertures should be at once cut in the walls.

Another expedient which increases the amount of fresh air to each man, is to disperse the patients, so as to have as few as possible in a ward; but, if, in spite of all feasable precautions, the disease makes its appearance, some decisive action must be taken. If no other method offers, the hospital must be evacuated, and the inmates be lodged in the open air, under trees and awnings, until, from improved circumstances, the epidemic is subdued.

Cleanliness is a thing which can only be attained in crowded hospitals by vigilant oversight of the surgeon, and hard work by his subordinates. Wounds will require dressing from one to three times a day, when actively discharging pus, and there is no way to accomplish the work, but by hard labor. It will generally be found best to avoid poultices, as they confine the pus too closely to the tissues; and to make a free use of water dressings. If there is any erysipelas or hospital gangrene, the same sponge should not be used upon two patients, as from its intricate structure it is impossible to cleanse it thoroughly, and it is liable to carry contagion. Many military surgeons exclude sponges from the hospitals entirely, and use pledgets of tow and cotton in their place, throwing them away at each dressing.

The surgeon also should bear in mind, that no probes or other instruments which have been used in a case of this disease, should ever be used on another patient, until they have been thoroughly cleansed by washing, and then dipped in boiling water. There is no doubt whatever, that some surgeons have carried death around their own hospitals, by their want of care in cleansing their instruments. In short, every precaution should be taken, which is required in the presence of a deadly contagious disease, which is capable of being propagated, both by inoculation and infection.

The curative treatment is both local and constitutional; the objects being to destroy the diseased tissue with its poisonous products, and to correct the abnormal diathesis from which it sprung.

The best local application is strong nitric acid. To apply it thoroughly, the sound integument around the slough must be coated with cerate, to protect it from cautery. If the slough is thick, it must be cut away with the scissors, and then the fuming acid applied by means of a pledget of lint tied to a stick, dipped in the acid and then pressed into every part of the wound. Chloroform may be given to obviate the pain. The acid converts the diseased tissue into a firm, hard coating, which, in a few days, sloughs off and leaves a healthy ulcer beneath. If, however, any part of the surface still appears diseased, the cautery must be repeated until normal granulations alone present themselves.

Some American surgeons in the Crimean war made use of creasote in the same manner, with excellent effect, and with less pain to the patient.

The constitutional treatment consists: first, in restoring the sick to a perfectly pure atmosphere and to entire cleanliness; and second, in the use of such tonics as will help them to resist the effects of the poison. The remedies which have the best effect for internal use, are quinine, nitric acid, and muriated tincture of iron. The latter is in many respects the most useful, because the system will tolerate it in larger quantities, without irritation. It should be given in doses of twenty or thirty drops every two hours, night and day.

Ammonia, which is often prescribed with a view to its stimulating effect, should be carefully avoided, because the system is already saturated with it, and ready to fall into dissolution from the deleterious influence of the alkaline diathesis.

### ERYSIPELAS.

This is another scourge of war surgery. It falls like a pestilence upon the wounded, carrying off hundreds of men, who, in civil surgery, might have recovered with ease.

The causes of erysipelas in the form seen in camp, are the same as those which produce hospital gangrene. Indeed I think that the poison is of the same nature only a little less powerful, so that it does not as promptly destroy the life of the tissue on which it lodges. All that I said, therefore, respecting the causes of hospital gangrene, may be applied without exception to the etiology of erysipelas.

It is unfortunate that the identity of several forms of this disease is so obscurely recognized by many writers. Thus, in a list of surgical cases, it is common to see one death ascribed to erysipelas, another to absorption of pus, or purulent infection, another to metastatic abscess, etc. Now all these are one and the same thing, and that thing is *erysipelas.* Pyæmia comes from aplastic phlebitis, and that form of phlebitis which produces pus in the veins and kills the patient, results always from the presence of the erysipelatous poison. A similar observation may truthfully be applied to the terms metastatic abscess and purulent infection. In fine, if your hospital is clean, the air pure, and the constitution of the men in a plastic diathesis, there will be very little erysipelas, phlebitis, pyæmia, absorption of pus, or metastatic abscess.

The symptoms of traumatic erysipelas are unfortunately too familiar to every surgeon. A few days after a surgical operation, perhaps a very brilliant and important one, a little puffy redness is seen upon the edges of the wound. The lymphatics leading from it, shoot red and tender streaks up to the nearest lymphatic glands. The glands thus injected with poison, become swollen and tender. The redness and swelling about the wound, extend rapidly, accompanied with a good deal of burning pain. The adhesive process in the wound is stopped, and an unhealthy aspect supervenes. Very often the limb affected swells enormously; the areolar tissue beneath the skin mortifies, and when exposed by an incision, presents the same gray gelatinous appearance which is observed in hospital gangrene. The skin itself being cut off from nutrition beneath, mortifies, and the extension of the disease often destroys the patient. After death, dissection brings to light, in the same patient, phlebitis, pyæmia, and metastatic abscesses. The constitutional symptoms are of the same adynamic typhous character as in hospital gangrene.

From what has been said, it is obvious that the prophylactic treatment, is chiefly good air and thorough cleanliness. The precautions before mentioned for avoiding contagion and infection, are also to be fully observed. In civil surgery, I have made extensive use, for three years, of muriated tincture of iron, as a prophylactic. Having observed the powerful effect of this remedy in cutting short the disease after it has commenced, it occurred to me to use it in advance, to prevent the attack. I have therefore given it with this view, after all my cutting operations, both in the hospital and in private practice, and the result is, that *since I have commenced this precaution, no patient of mine has died of phlebitis, pyæmia, nor any other form of traumatic erysipelas.* I cannot but think, therefore, that the adoption of the same plan would greatly reduce the mortality in military hospitals. But this medication is by no means a substitute for thorough ventilation and cleanliness. If the fetid gases and foul discharges of a thousand wounds are allowed to saturate the blood of the hospital inmates with poison, let no surgeon think that he can evade all the consequences by pouring into their rotten systems, any medical preservative. Some, it is true, may be cured even then, but very many will die.

The curative treatment of erysipelas is now pretty well settled. Taking as a clue, the fact that it is a disease of an aplastic nature, in which an excess of alkaline secretions is present, tending by that excess to dissolve and break down the tissues, we readily see why all experience points to the use of such remedies as have the power of destroying alkaline products. By far the most efficient remedy in erysipelas, is per-chloride of iron, usually given in the form of the muriated tincture. Per-chloride of iron is an unstable compound, and presented to any alkaline fluid, it immediately neutralizes it by yielding a large quantity of chlorine. Hence, its prodigious power of reducing to a solid form, various alkaline fluids of the body, such as pus, blood, and serum. When given freely, it is capable of completely changing the diathesis of a patient within thirty hours from a purulent to a plastic condition. Most striking illustrations of this fact have occurred in my practice.

Two pathological conditions are necessary to produce an erysipelas. One is an alkaline, and therefore aplastic diathesis, marked by a peculiar tendency to produce serum, or pus upon the slightest irritation, and by a striking incapacity to limit its spread by any plastic barriers. The other condition is the presence of a specific poison of an irritant character, capable of inoculation, and which produces the inflammatory action. If both these conditions be present, the patient has erysipelas; if either be absent the disease cannot exist. The per-chloride of iron acts directly, I think, upon both conditions. It rapidly neutralizes the alkalies and induces a plastic diathesis, and the chlorine destroys the organic poison. In order to get this decisive influence, it is not enough to give ten or fifteen drops of the tincture a few times a day. Twenty or thirty drops must be given every hour or two, until the effect is produced. The extreme rapidity of the disease requires the utmost promptitude of action.

Other tonic remedies have also a good reputation, especially quinine given in large and frequent doses. The mineral acids are also useful, but the vegetable acids seem to be inert when given internally, because being organic, they are digested and destroyed in the stomach.

For local treatment, one of the most useful things is to make free incisions for the evacuation of the poisonous serum and pus. Next to incision, the abundant application of iodine or nitrate of silver is valued by our authorities. If iodine is chosen, it should be dissolved in glycerine, and kept constantly applied. The painting of the surface with the tincture, lacks efficiency, because the alcohol evaporates in a few minutes, leaving the iodine dry and in an unfavorable form for absorption. In domestic practice, the cranberry poultice is often used with good effect, the acid of the berry serving to neutralize the alkali of the diseased part.

Cold water is much less efficient in this disease than in simple inflammation, and poultices, unless of some acid or astringent substance are injurious.

In general the best treatment is a very free use of quinine and tincture of iron internally, and a few incisions, with glycerine and iodine externally.

